# Identification of a Novel Sulfonamide Non-Nucleoside Reverse Transcriptase Inhibitor by a Phenotypic HIV-1 Full Replication Assay

**DOI:** 10.1371/journal.pone.0068767

**Published:** 2013-07-18

**Authors:** Tae-Hee Kim, Yoonae Ko, Thierry Christophe, Jonathan Cechetto, Junwon Kim, Kyoung-Ae Kim, Annette S. Boese, Jean-Michel Garcia, Denis Fenistein, Moon Kyeong Ju, Junghwan Kim, Sung-Jun Han, Ho Jeong Kwon, Vincent Brondani, Peter Sommer

**Affiliations:** 1 Institut Pasteur Korea, Seongnam-Si, Gyeonggi-Do, Korea; 2 Department of Biotechnology, Translational Research Center for Protein Function Control, College of Life Science and Biotechnology, Yonsei University, Seoul, Korea; CHA University, Republic of Korea

## Abstract

Classical target-based, high-throughput screening has been useful for the identification of inhibitors for known molecular mechanisms involved in the HIV life cycle. In this study, the development of a cell-based assay that uses a phenotypic drug discovery approach based on automated high-content screening is described. Using this screening approach, the antiviral activity of 26,500 small molecules from a relevant chemical scaffold library was evaluated. Among the selected hits, one sulfonamide compound showed strong anti-HIV activity against wild-type and clinically relevant multidrug resistant HIV strains. The biochemical inhibition, point resistance mutations and the activity of structural analogs allowed us to understand the mode of action and propose a binding model for this compound with HIV-1 reverse transcriptase.

## Introduction

Since the first description of the acquired immunodeficiency syndrome (AIDS) in 1981, the human immunodeficiency virus (HIV)/AIDS pandemic has spread throughout the world, killing half of the 60 million people infected thus far [1], [2]. Currently, more than 30 million people are living with HIV/AIDS worldwide, and about 2.5 million new HIV infections occur each year. As a consequence of the development and availability of effective antiretroviral therapy for HIV infection, certainly a major achievement of recent medical research, the number of people dying of AIDS-related causes is presently however in decline [2]. Anti-HIV medications currently approved by the U.S. Food and Drug Administration fall into six therapeutic classes: (i) Nonnucleoside Reverse Transcriptase Inhibitors (NNRTIs), (ii) Nucleoside Reverse Transcriptase Inhibitors (NRTIs), (iii) Protease Inhibitors (PIs), (iv) Fusion Inhibitors, (v) Entry Inhibitors and (vi) Integrase Inhibitors [3]. The recommended treatment strategy - Highly Active Antiretroviral Therapy - combines three or more anti-HIV medications in a daily regimen and has considerably improved the quality of life for infected people by delaying the progression of the disease and reducing disabilities, making HIV/AIDS a chronic disease, not a death sentence [3], [4]. However, these medications do not cure HIV infection, and individuals taking these medications can still transmit HIV to others [4], [5], [6], [7], [8]. Moreover, complex dosing, drug-drug interactions and toxicities restrict many of these regimens. Adverse drug side effects, which are frequently associated with long-term treatment, and the rapid increase of viral strains that are resistant to available antiretroviral drugs threaten the success of current HIV treatment, emphasizing the importance of developing alternative anti-HIV compounds [3], [4], [9], [10].

Various screening approaches such as virus-based assays, structure-based drug design, receptor pharmacology and biochemical screening, are currently available for HIV drug discovery. Several of these approaches have been used successfully to identify new HIV inhibitors. In addition, a variety of cell lines and reporter assays that are suitable for cell-based high-throughput screening (HTS) assays to various degrees have been established [11]. Many of the described cell-based HIV antiviral assay formats utilize either reporter virus or a reporter cell line to measure viral replication. In reporter virus assays, cells are infected with a recombinant reporter virus, and viral replication is quantified by measuring the expression of the reporter gene. In reporter cell assays, the target cells are engineered to contain a reporter gene that is activated upon viral infection. In this case, virus replication is measured by monitoring the induction of the reporter gene after infection. Cell-based screening approaches are advantageous because they can include multiple targets and toxicity in a single screen and include targets and/or structures that are not amenable to biochemical screening methods [11].

Herein, we describe the development and validation of a phenotypic, HIV-1 full replication assay based on cells harboring the green fluorescent protein (GFP) reporter under the control of the HIV-1 promoter [12], [13]. The cells show very low basal GFP expression, but infection with HIV-1 induces GFP due to the presence of the viral transactivator Tat. All steps of the infection and replication process can be targeted in a chemical screen. Implementation of this HTS assay in a screen of 26,500 molecules enabled us to identify an interesting sulfonamide chemical scaffold with high anti-HIV-1 activity. Here, we present the study of this scaffold including its putative mode of action at the molecular level.

## Materials and Methods

### Disposable Materials

Cell culture flasks (T75 and T175) were obtained from BD/Falcon (California, USA). Syto60 was purchased from Molecular Probes/Invitrogen (Carlsbad, CA). The small molecule library was formatted in 96- and 384-well polypropylene plates from Greiner (Frickenhausen, Germany). Clear plastic bottom 384-well Evotec plates (Frickenhausen, Germany) were used as assay plates for imaging.

### Cells, Cell Culture and Virus Production

CEMx174-LTR-GFP CG8 cells, a clonal derivative of CEMx174 cells transduced with HIV-based retroviral vector particles encoding GFP under the control of the HIV-1_LAI_ LTR, and HeLa-derived reporter cells (HeLa-LTR-GFP cells) were established and cultured as previously described [12], [13]. All cell culture reagents and media supplements were obtained from GIBCO (Carlsbad, CA). HIV-1_LAI_ and HIV-1_RTMDR1_/MT-2 (M41L, L74V, V106A, T215Y resistant to AZT, ddI, nevirapine, delavirdine and efavirenz) stocks were produced in CEMx174-LTR-GFP CG8 cells. Infected cells were cultured in complete RPMI medium, and virus-containing supernatants were harvested at the peak of virus production (5 days). The culture fluids were filtered (0.45 µm), and aliquots were stored at −80°C. Virus stocks were titrated by quantification of HIV-1 p24 core antigen levels using the Alliance HIV-1 p24 ELISA kit (Perkin-Elmer) according to the manufacturer’s instructions. TCID50 values were determined by serial dilution of virus stock supernatant, and triplicate infection was performed. Values were calculated using the Reed and Muench formula [14].

The virus used in the Monogram experiment was a pNL4-3 derivate with point mutations K103N (resistant to nevirapine), V106A (resistant to nevirapine), Y181C (resistant to nevirapine, delfinavir), double mutant K103N/Y181C (nevirapine, delfinavir and efavirenz) and multidrug resistance mutant MDRC4 (with reduced susceptibility to all licensed reverse transcriptase and protease inhibitors).

### Chemical Library and Compound Management

A library of 26,500 small synthetic molecules was purchased from TimTec (Newark, USA). It is composed of 25,000 compounds from the ActiProbe diverse library, 1,000 compounds from a kinase inhibitor library (ActiTargK) and 500 compounds from a protease inhibitor library (ActiTargP). The library was received as dry powders, suspended in dimethyl sulfoxide (DMSO) at a concentration of 10 mM and transferred into 96-well polypropylene master plates (Greiner-651201). For high-throughput screening, compounds were reformatted in DMSO at a final concentration of 2 mM into 384-well compound plates (Greiner-781280) using an automated liquid handler (Agilent Benchcel). Those plates were kept frozen until use. Immediately before a screening campaign, compound plates were thawed at room temperature and 250 nL of the compounds was transferred directly into the wells of assay plates containing 10 µL of PBS/well using an Evobird liquid handler (Evobird). The final DMSO concentration in the assay was 0.5%. A fast mixing was performed to ensure a complete diffusion of the small volume of stock compounds in the assay wells. Reference compounds (AZT, nevirapine and saquinavir; all were obtained through the AIDS Research and Reference Reagent Program, Division of AIDS, NIAID, NIH) were prepared in 10 mM stock solutions. All screening procedures were performed in a biosafety level 3 facility.

### High-throughput Screening

Twenty-five microliters of CEMx174-LTR-GFP CG8 cells (4,000 cells/well) in cell culture medium was plated into a 384-well format with pre-dispensed compounds, using a microplate dispenser (Matrix WellMate; Thermo Scientific). Plates were incubated for 1 h at 37°C, 5% CO_2,_ and 15 µL of virus-containing supernatant (multiplicity of infection M.O.I. = 3) was added using a Matrix WellMate. Positive (10 µM nevirapine) and negative (0.5% DMSO) controls were included in 16 wells each for every plate. Assay plates were further incubated for 5 days at 37°C, 5% CO_2_, until readout. After the addition of 10 µL of 5 µM Syto60 in each well for nuclear counterstaining and incubation for 1 h at room temperature, images were acquired with an automated confocal microscope (Opera; PerkinElmer) using a 20X water objective (NA 0.70), 488 nm and 635 nm lasers and a 488/635 dichroic mirror. Three image fields were recorded for each well. Image analysis was then done using PE Accapella software. The algorithms first identify the nuclei stained by Syto60 in the red 635 nm channel. The number of red delineated surfaces corresponds to the number of cells counted in the analysis. The cell is then considered to be infected by virus if there is an overlap of the signal from the green channel. The ratio of infected cells to the total number of cells determined the percentage of infected cells.

Data obtained from image analysis were then analyzed using IDBS ActivityBase to calculate the percentage of inhibition, the screening quality (Z score) and the assay quality (Z’ with a coefficient of variation), which were calculated as defined by Zhang, et al [15]. Dose-response curve fitting and IC_50_ values were calculated using GraphPad Prism 4 (GraphPad Software, Inc).

### Hit Confirmation

To confirm the activity of identified hits, each selected compound was tested in a 10- point dose-response with 3-fold serial dilutions (from 10 µM to 0.25 nM) in duplicate using the CEMx174-LTR-GFP CG8 cell-based imaging assay.

### Cell Viability Assay

Cytotoxicity of the selected hits was determined using the same cell lines (CEMx174-LTR-GFP CG8 cells and HeLa-LTR-GFP cells) plated in Evotec 384-well plates with pre-dispensed compounds but without viral infection. After the 5th day of incubation, cell viability was evaluated using 100 µg/mL of resazurin (Sigma, St. Louis, MO). After 2 h of incubation at 37°C, 5% CO2, the fluorescence of resorufin/resazurin was measured at 544 nm/595 nm (excitation/emission) on a PE Victor 3 plate reader.

### P24 Core ELISA

Extracellular p24 was measured using the Alliance HIV-1 p24 ELISA kit (Perkin-Elmer) according to the manufacturer’s instructions. Cell-free supernatant from infected cultures was harvested and stored at −80°C prior to quantification. CEMx174 cells were incubated for 1 h with 5 µM IPK1 and nevirapine compounds prior to infection with wild-type NL4-3 HIV-1 virus (MOI = 3). Aliquots of medium were taken every 3 or 4 days. Medium was adjusted by adding fresh compound for a final concentration of 5 µM, and the p24 level was determined.

### Monogram

Monogram Biosciences' PhenoScreen™ is a phenotypic screening assay designed to evaluate the activity of reverse transcriptase. The viruses used to perform the assay were wild-type, multidrug resistant and the single point mutations: K103N, Y181C, and V106A and the double mutation K103N/Y181C. The experiments were performed as described in [16].

### Reverse Transcriptase *in vitro* Enzymatic Assay

Reverse transcriptase activity was measured using the Reverse Transcriptase assay colorimetric kit (Roche) according to the manufacturer’s instructions.

### Computer Modeling

RT/TMC125 co-crystallography structures were downloaded from the Protein Data Bank (HXB2: HIV-1 group m subtype b, 3M8P: wild type, 3MED: K103N mutant, and 3DRR: Y181C mutant) [17], [18], [19], [20]. Only the RT protein structure was used for docking, and all water molecules and TMC 125 were removed [21].

The ligand-receptor docking was performed using Glide, Schrödinger, LLC. [22]. Docking grids were generated with a size similar to the original ligand. TMC-125 was docked as a reference molecule to determine the quality of the docking results. We used the XP (extra-precision) mode, which provides a more discriminating determination of unfavorable energy for the docked structure. The potential energy with the binding molecule was calculated by MacroModel, Schrödinger, LLC. [23]. The OPLS_2005 parameter was applied for all procedures.

## Results

### Development of a Phenotypic High-throughput HIV Full Replication Assay

**Figure 1:**
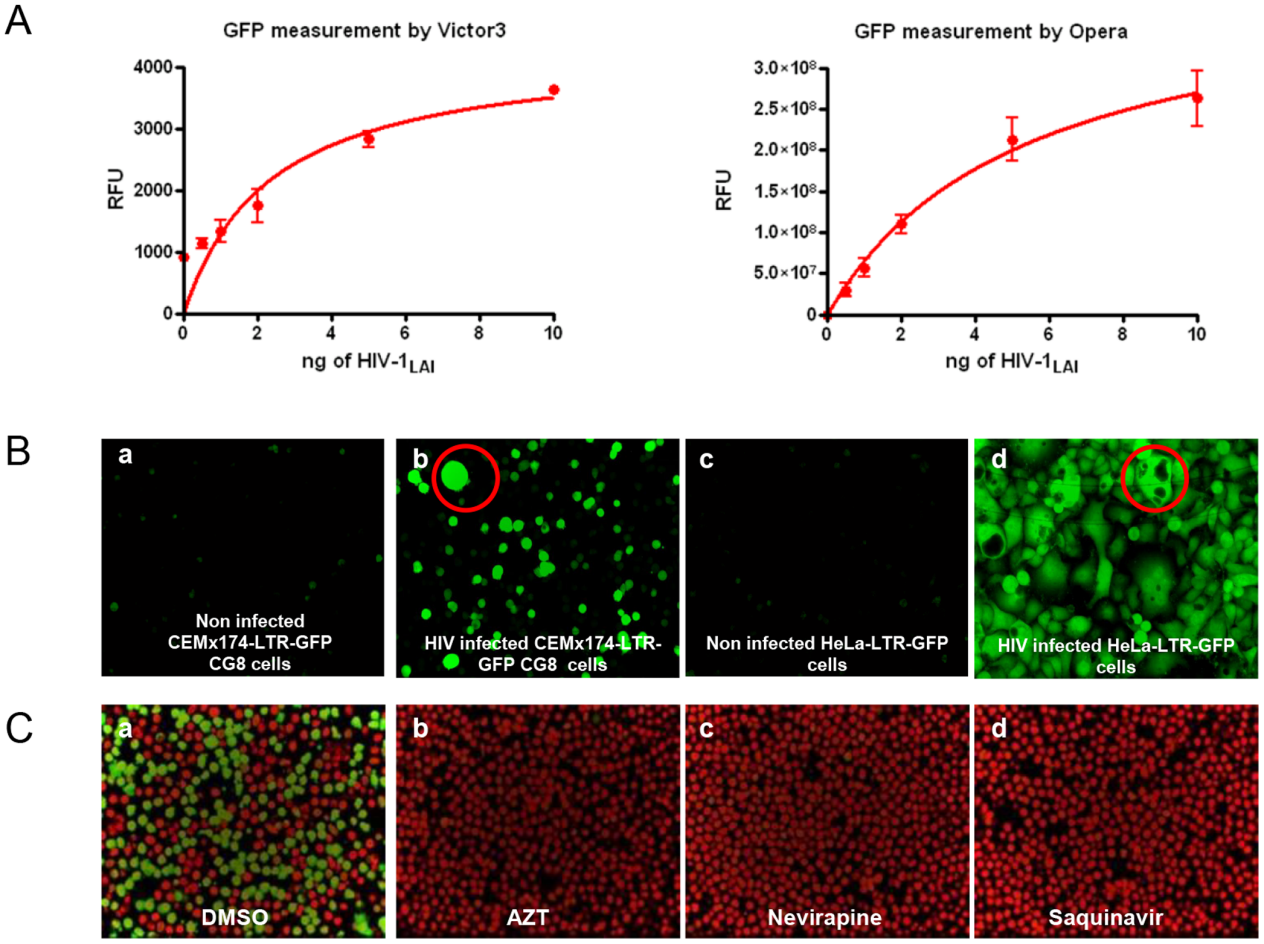
HIV infection assay development. A. Measurement of GFP reporter activity, using Victor 3 and Opera reader, in CEMx 174-LTR-GFP CG8 cells upon HIV-1_LAI_ infection. B. Infection of CEMx 174-LTR-GFP CG8 and HeLa-LTR-GFP cells: Opera-acquired images showed infected (Panel b and d), unlike uninfected (Panel a and c) cells, carried the GFP reporter gene. Syncytia are indicated by red circles. C. Assay validation upon antiretroviral treatment: reference drugs were tested to evaluate the assay performance using an Opera reader. CEMx 174-LTR-GFP CG8 cells infected by HIV-1_LAI_ were treated with AZT (Panel b), nevirapine (Panel c) and saquinavir (Panel d). Images show nucleus detection in red and infected cells in green (Panel a: DMSO).

To develop a phenotypic, high-throughput HIV full replication assay, we used CEMx174-LTR-GFP CG8 reporter cells. These cells carry an integrated HIV mini-genome encoding the GFP gene under the transcriptional control of the genuine HIV-1_LAI_ LTR promoter [13]. The cells show minimal levels of basal GFP expression in an uninfected state but respond to HIV infection with the induction of GFP fluorescence, as depicted in Figure 1. To facilitate effective screening under high-throughput conditions, we initially optimized the cell density (4,000 cells/well), the amount of virus used for infection (10 ng p24/well) and the incubation time (5 days) (data not shown). Furthermore, we tested DMSO tolerance because this typical carrier substance is used during drug screens. By testing the effect of different DMSO concentrations on cell viability and HIV infection, we determined that final DMSO concentrations up to 0.5% were optimal, while concentrations higher than 1% diminished cell viability and HIV infection rates (data not shown). Finally, we also compared two different assay read-outs, the measurement of total fluorescence intensities using a fluorescence plate reader (Victor 3) and automated confocal microscopy (Opera) followed by advanced image analysis. Identical results were obtained with both methods (Fig. 1A). The visual readout has the advantage of including information concerning the number and viability of cells by using cellular staining before image acquisition. In addition, the syncytia formation can be observed when the cells are infected by HIV-1_LAI_ in both cell lines, the CEMx174-LTR-GFP CG8 (Fig. 1B, suspension cell, panel b) and HeLa-LTR-GFP (Fig. 1B, adherent cell, panel d) cell lines.

### Validation of the Assay with Known Anti-HIV Drugs

To evaluate whether the CEMx174-LTR-GFP CG8 cells could be used to screen for various classes of HIV inhibitors, we tested the antiviral activity of the established HIV-1 reverse transcriptase inhibitors AZT (NRTI) and nevirapine (NNRTI) and the HIV-1 protease inhibitor saquinavir. For this purpose, all drugs were titrated over a wide concentration range onto CEMx174-LTR-GFP CG8 cells. The anti HIV-1 drugs were preincubated with the cells prior to HIV-1_LAI_ infection. These conditions were suitable to detect compounds that interfere with all HIV life cycle steps. The infection was performed at a multiplicity of infection (M.O.I.) of 3, which corresponds to 10 ng of HIV-1_LAI_. This procedure allowed infections to proceed for prolonged times and enabled the infection to spread without a significant cytopathic effect. As shown in Figure 1C, treatment with the three reference drugs resulted in HIV-1 inhibition. The determined IC_50_ values were characterized and were comparable to previously published data [24], [25], [26]. This experiment confirmed that the cell-based screening system was robust and compatible to further adaptation to High-Content/High-Throughput Screening.

### Primary High-throughput Screening and Hit Confirmation

The validated CEMx174-LTR-GFP CG8 cell-based HIV full replication assay was used to screen a library of 26,500 small molecules. Among the hits (selection criteria: 70% inhibition), 100 compounds exhibited less than 50% cytotoxicity (hit rate 0.37%). As an internal control, we found three known anti-HIV inhibitors: colchicine, an inhibitor of microtubule polymerization and a weak inhibitor of HIV-1 associated with moderate cytotoxicity, floxuridine and 5-fluorouridine, both inhibitors of thymidylate synthase.

The activity of those primary hits was then confirmed in a 10-point dose response with 3-fold serial dilutions starting at 10 µM (data not shown). In the confirmed hits, we found 34 anti-HIV compounds with an IC_50_<1 µM that showed concentration-dependent inhibition with well-fitted curves. In addition to potency, the cytotoxicity of the selected hits was also assessed in both imaging assay and resazurin-based assays for confirmation.

### Characterization of a Highly Active Non-Nucleoside Reverse Transcriptase Inhibitor (NNRTI)

**Figure 2:**
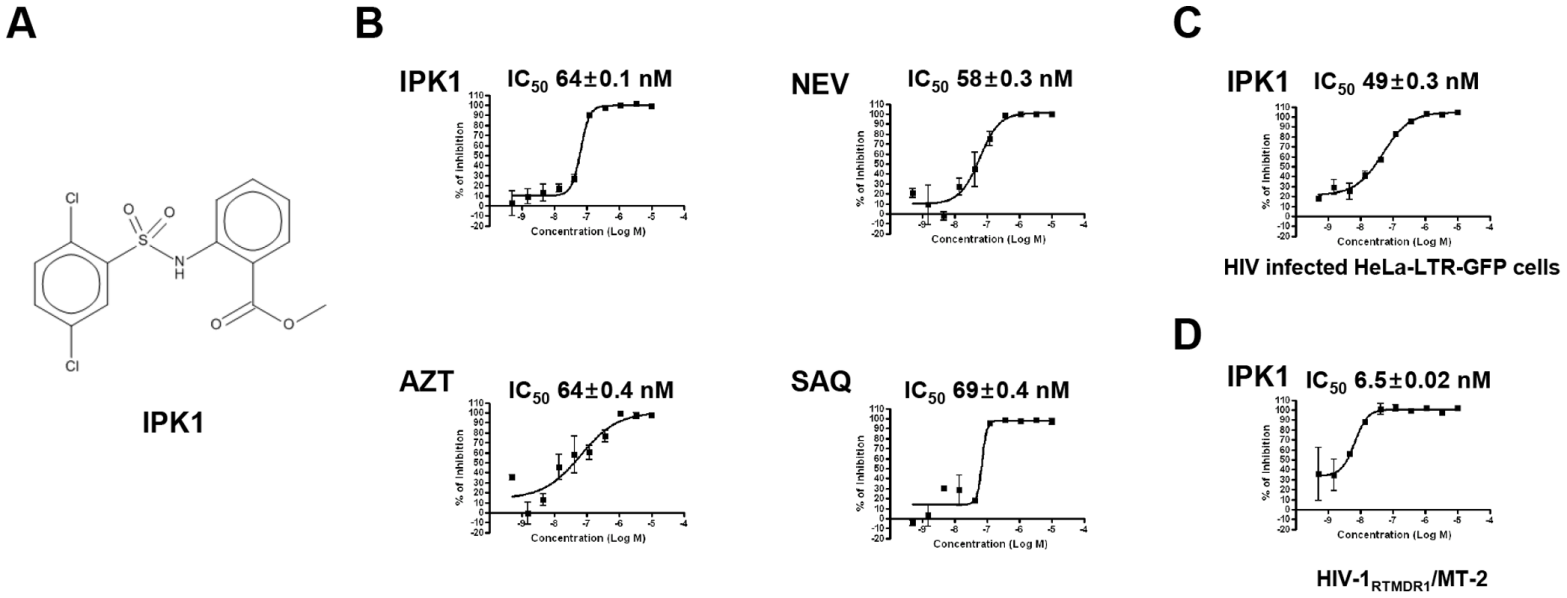
Identification of IPK1 as a potent antiretroviral hit compound. A. Chemical structure of the **IPK1** compound. B. Dose-response curve of **IPK1** and comparison to reference anti-HIV drugs. The IC_50_ value was characterized in CEMx 174-LTR-GFP CG8 cells infected by HIV-1_LAI_. C. **IPK1** activity defined by IC_50_ characterization in HeLa-LTR-GFP cells upon HIV-1_LAI_ infection. D. **IPK1** activity against the multidrug resistant virus HIV-1_RTMDR_/MT-2 in CEMx 174-LTR-GFP CG8.

Based on this screen, we identified one potent compound scaffold, referred to hereafter as **IPK1** (Fig. 2A), that exhibited activity against HIV-1_LAI_ infection in CEMx174-LTR-GFP CG8 cells similar to the reference drugs. This chemical structure, which contains a sulfonamide group, is active at low nanomolar concentrations (IC_50_ of about 64 nM), similar to that of the characterized HIV reference compounds nevirapine, AZT and saquinavir (Fig. 2B). In addition, the compound does not exhibit cellular toxicity up to 10 µM (selectivity index >200, Fig. S1.) and has the same potency and lack of cytotoxicity in the HeLa-LTR-GFP cellular model (Fig. 2C). The **IPK1** activity was assessed in a p24 assay to confirm that this molecule did not interfere with the reporter itself. Antiviral activity was observed under this experimental condition (Fig. S2). To further characterize the compound, we tested its activity in an assay with the multi-drug reverse transcriptase (RT) resistant virus HIV-1_RTMDR1_/MT-2. The obtained results (Fig. 2D) showed that the **IPK1** compound inhibited HIV-1_RTMDR1_/MT-2 with a high potency (IC_50_ around 6 nM), which reflects an attractive feature for anti-retroviral active compounds.

To further analyze the activity of **IPK1**, we tested its activity against a panel of classical RT point resistance mutations such as K103N, Y181C and V106A. The results were obtained using Monogram Biosciences' PhenoScreen™ assay. The IC_50_ values presented in Table 1 indicate that although the reference control TMC125 [25] was not affected by these mutations, the compound **IPK1** showed a differential activity. The **IPK1** compound was active against the clinical isolate RT mutant MDRC4 and the V106A point mutation, but it had lower activity with the K103N and Y181C mutations. A moderate resistance was observed for the K103N mutation, with an IC_50_ ratio (Mutant/WT) of 71, whereas complete resistance was observed for the Y181C and the double mutant K103N/Y181C mutant (IC_50_ ratio >200). Together, these observations suggested that the compound targets the RT enzyme.

**Table 1:** IC_50_ characterization of IPK1 and TMC125.

	IC_50_ (nM)	
HIV strains	TMC125	IPK1
WT	1.4	24
MDRC4	6.8	199
K103N	1.8	1777
Y181C	5.9	>10000
K103N/Y181C	19.3	>10000
V106A	1.3	1.6

Antiviral activity of **IPK1** and TMC125 compounds against defined reverse transcriptase resistant mutations using the Monogram Bioscience experimental assay.

**Figure 3:**
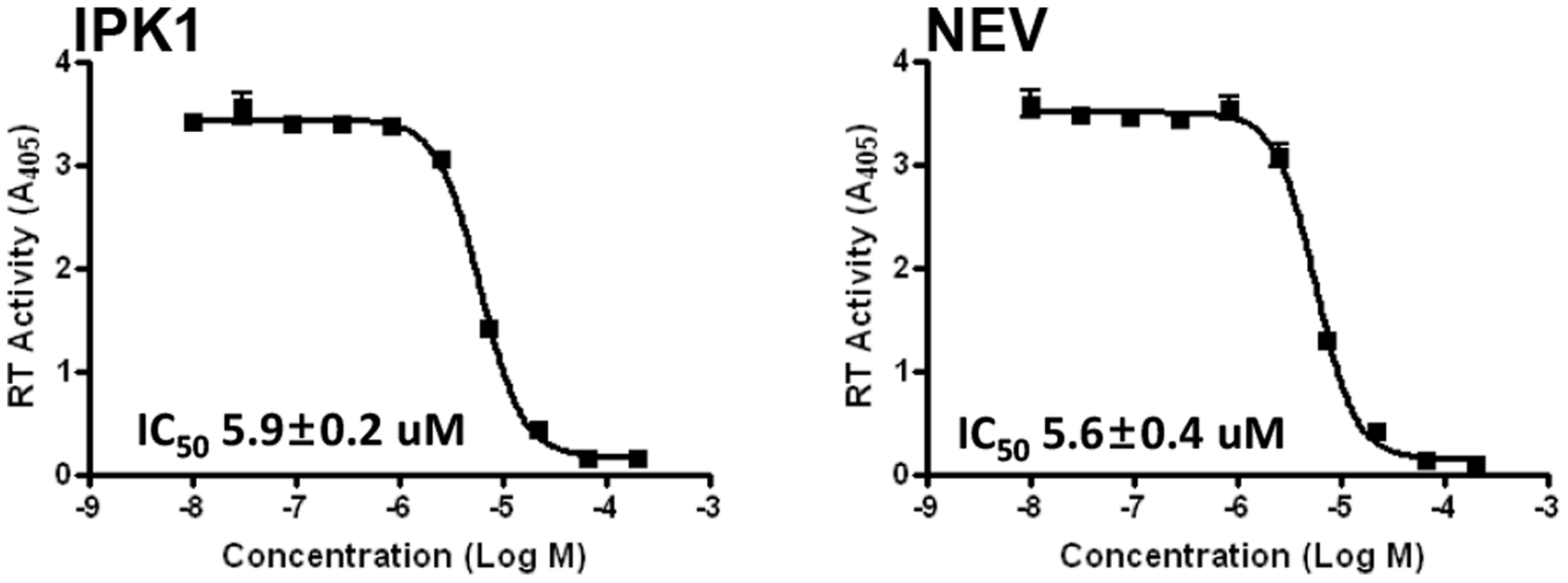
Anti-reverse transcriptase activity of the IPK1 compound and nevirapine. The IC_50_ value was determined *in vitro* using purified enzyme.

To confirm this hypothesis, we performed an *in vitro* biochemical experiment using purified RT enzyme. The results depicted in Figure 3 show the data obtained with nevirapine as a positive control and with **IPK1**. The calculated IC_50_ values for the two molecules were identical and corroborated the observed resistance mutation profile. In addition, kinetic experiment was performed with IPK1 and nevirapine shows identical profile correlating with the pattern of noncompetitive inhibitor (Fig. S3).

### Computer Modeling of the IPK1 Interaction with HIV-1 Reverse Transcriptase

The NNRTI binding mode has been well defined by many co-crystallography studies [18], [19], [20]. The binding mode of TMC125, which is a well-known second generation NNRTI, is also defined from crystallography [18]. The key residues were described, including the hydrogen bond interaction with K101 at 2.5 Å and hydrophobic interactions with Y181, Y188, W229 and F227. We used a docking method to model the **IPK1** interaction with reverse transcriptase at the NNRTI-relevant residues.

**Figure 4:**
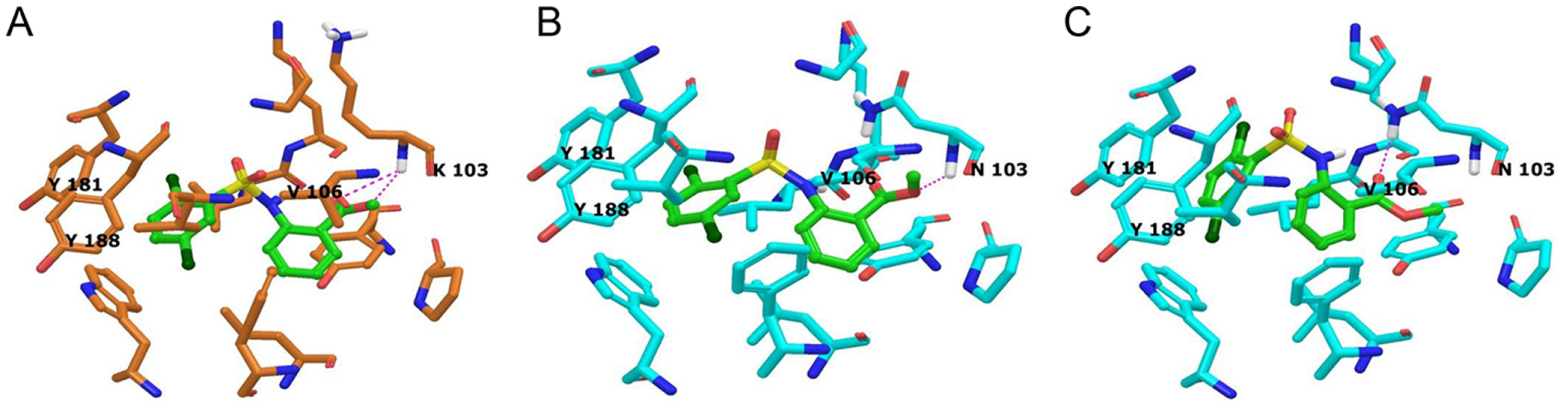
Docking of the IPK1 compound against HIV-1 reverse transcriptase. Both wild-type (Panel A) and a K103N mutant (Panel B and C) are shown. Hydrogen bonds are marked as pink dotted lines. The total energies of the reverse Transcriptase are −7337.9 kJ/mol, −6614.5 kJ/mol and −6826.3 kJ/mol, respectively.

The docking of **IPK1** was performed in HIV-1 RT wild-type and drug-resistant mutant structures to examine its correlation with the biological activity that was measured in parallel. From the WT docking result, the dichlorophenyl group was positioned within the hydrophobic pocket of the NNRTI binding site. It was surrounded by Y181, Y188, W229 and F227, which have been defined as hydrophobic residues involved in a *π-π* interaction. Interestingly, the acetate of the methyl benzoate moiety made possible two hydrogen bond interactions with the K103 backbone within 3.5 Å (Fig. 4, Panel A). The docking results, obtained with the point mutations K103N and Y181C, could be correlated with **IPK1’s** biological activity. In fact, we were not able to calculate a stable interaction with the Y181C mutant (high resistance score), whereas a docking with the K103N mutant (low resistance score) was obtained. Two possible models are presented (Fig. 4, Panel B and C) in which the hydrogen bond interaction with the K103N mutation is retained. The lower **IPK1** biological activity obtained with K103N, compared to WT, can be explained by this weaker hydrogen bond interaction. In both K103N interaction models, the docking with the hydrophobic residues showed two possible conformations, one identical to the interaction obtained with the wild type (Fig. 4, Panel B) and a second that was different but more stable (Fig. 4, Panel C). Altogether, the biological activity of **IPK1** on WT and several NNRTI-resistant mutants was in good agreement with the docking scores (WT: −8.65, K103N: −5.25 and Y181C: none). Considering that TMC-125 had a docking score of −13.74 and −13.68 in WT and K103N, respectively, the docking results also matched the biological assay results. For both docked structures, the calculated energies were −7337.9 kJ/mol for WT and −6614.5 kJ/mol and −6826.3 kJ/mol for the two K103N docked poses. For Y181C, a stable binding mode could not be found for **IPK1** and we were not able to calculate the binding energy.

**Figure 5:**
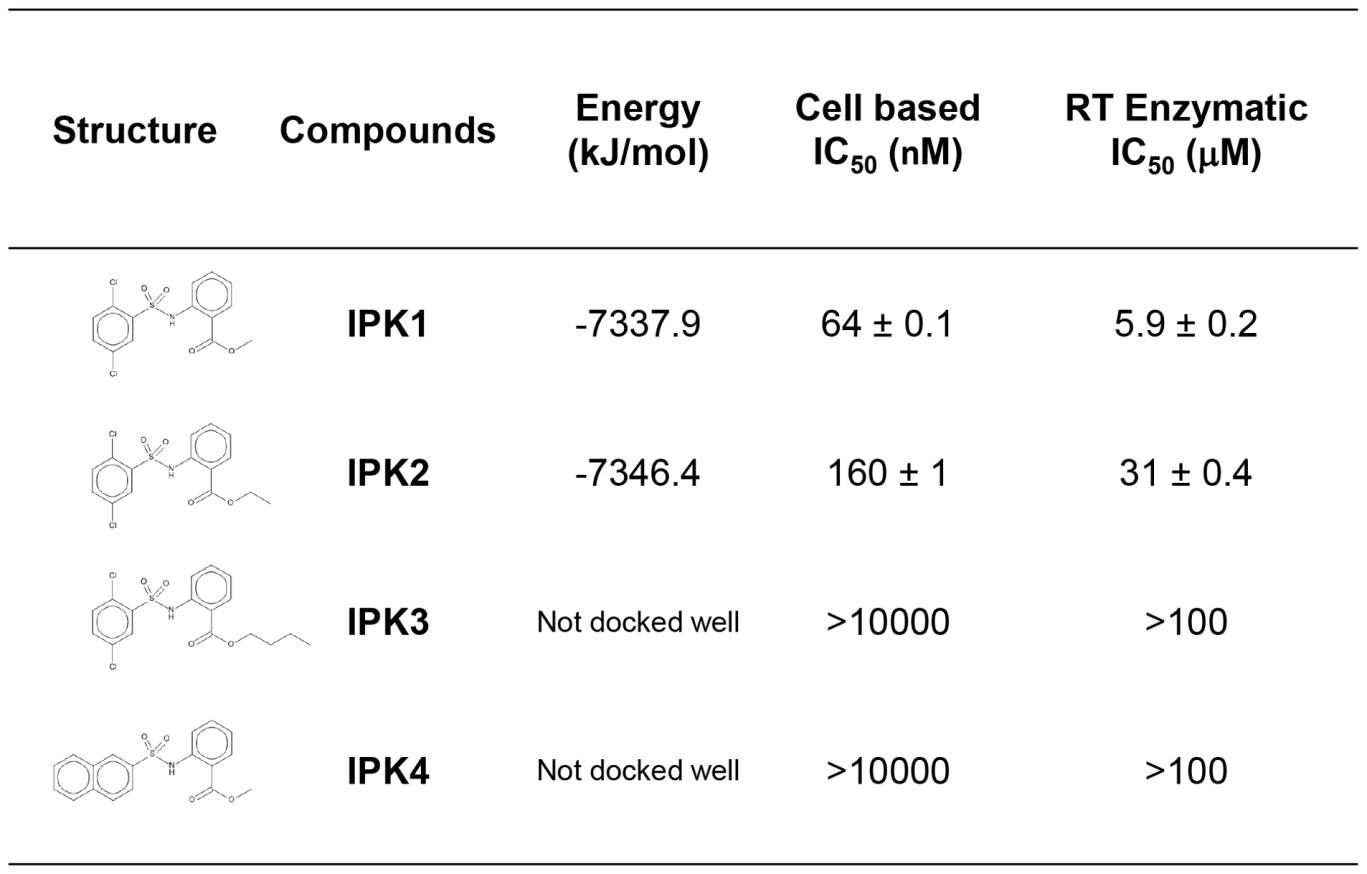
Table of calculated docking energy and IC_50_ characterization of the IPK1 compound and analogs.

To further analyze this binding mode, we tested the activity of several **IPK1** related analogs designed based on their ability to be docked or not in this model. For the benzoate moiety, we tested the effect of the length of alkyl ester chain: methyl (**IPK1**, ClogP = 4.17), ethyl (**IPK2**, ClogP = 4.70) and n-butyl (**IPK3**, ClogP = 5.76). Only a short extension (methyl to ethyl) could be accommodated, and longer chains destabilized the interaction, as predicted by the docking score of −7337.9 kJ/mol for **IPK1** and −7346.4 kJ/mol for **IPK2** and the lack of a stable form for **IPK3** (Fig. 5). The inhibitory activity observed with the cell based assay correlates with the docking results. In fact, only **IPK1** and **IPK2** were able to inhibit viral replication (IC_50_ of 64 and 160 nM respectively); **IPK3** was inactive. On the other side of the **IPK1** molecule, we tested the substitution of the dichlorophenyl group by 2-naphtyl (**IPK4**, ClogP = 4.08), which is a larger aromatic structure. We were not able to obtain any stable docking model for this structural modification, and this analogue did not exhibit any HIV inhibitory activity. In addition, the RT biochemical inhibitory activity of **IPK1** compounds and analogs were assessed. Analysis of those molecules in cellular and RT enzymatic experiments showed similar activity (Fig. 5). Only **IPK1** and **IPK2** exhibited antiviral activity and RT inhibition.

Together, the docking and inhibitory activity results obtained with the **IPK1** analogs corroborate the computer modeling interaction of this molecule and confirmed that the observed **IPK1** compound activity is associated with RT inhibition.

## Discussion

Here we report a screening method based on a cellular phenotypic assay utilizing image analysis. Using a well-defined experimental setup, this procedure was suitable to characterize active molecules against any steps of the HIV-1 life cycle. When comparing our developed technology to others, based on viral cytopathogenicity, this high-content approach allows visualization of compound activity while simultaneously assessing cellular toxicity in a single assay. Furthermore, due to the microscopy readout, additional biologically relevant markers could be incorporated toward high-content screening. This strategy may be successfully extended to develop screening assays for inhibitors of other viruses.

Starting from a relatively small compound library of both diverse and focused scaffolds, we were able to identify hits and find a unique potent compound, **IPK1**, that does not exhibit cellular toxicity. Further analysis of this biologically active chemical was performed, and we identified its properties. The antiviral activity of the **IPK1** compound was in the nanomolar range, which is comparable with known antiviral molecules available on the market. In addition, clinical isolated reverse transcriptase mutants (HIV-1_RTMDR1_/MT-2 and MDRC4) were also inhibited in *in vitro* replicative assays. Finally, we tried to elucidate the mode of action of this compound by testing classical resistance mutations against NNRTI molecules and biochemical properties.

According to the resistance profile obtained with point mutations (K103N and Y181C), a relevant computer modeling was performed. The docking of **IPK1** and analogs in the NNRTI pocket correlated with the *in vitro* assay data. The interaction model corroborates the binding of TMC125 (−7515.9 kJ/mol) and additional reference compounds such as nevirapine and MK4965 (−7132.7 kJ/mol and −7725.3 kJ/mol, data not shown). One of the main differences between those co-crystal structures is the involvement of the K103 residue that interacts with nevirapine. In both cases, modeled interactions of **IPK1** had a similar binding mode in the pocket of the wild-type reverse transcriptase. Based on the TMC125/K103N mutant co-crystal structure, we were able to model the interaction of **IPK1** with the K103N mutant that correlates with the observed resistance level *in vitro*. We did not observe resistance induced by the mutation V106A, which also corroborates the *in silico* **IPK1** binding mode. In addition, we were able to examine the effect of some chemical modifications (analogs **IPK2**, **IPK3** and **IPK4**) that resulted in the loss of biological activity in agreement with the predictions from the docking model. Other groups have identified active HIV-1 reverse transcriptase inhibitors containing sulfonamide [27], [28], [29] that were structually different from **IPK1**. The synthesis of **IPK1** is straightforward via a coupling reaction between 2,5-dichlorobenzene sulfonyl chloride and methyl 2-aminobenzoate in pyridine to give a sulfonamide product with good yield [unpublished data]. This compound could lead to an antiretroviral drug accessible to the vast majority of people infected with HIV-1 living in developing countries that presently have limited access to current treatment. The good correlation between our modeling and the *in vitro* experimental data suggests that these methods could be used in rational design of new molecules derived from the reaction of various sulfonyl chlorides and amines to improve the antiviral activity of this series against WT and mutant HIV-1.

## Supporting Information

Figure S1 **Determination of cellular toxicity by nucleus counting.** Cells were incubated with several concentrations of **IPK1**, AZT, nevirapine, and saquinavir compounds. The nuclei of cells were stained by Syto60, and images were analyzed by Accapella Software.
(TIF)

Figure S2 ***In vitro*** **inhibition of viral replication.** Antiviral **IPK1** and nevirapine activity was analyzed using a p24 ELISA assay by measurement at day 3, 7, 10, 14 and 17 post-infection.
(TIF)

Figure S3 **Kinetics of reverse transcriptase activity.** Reverse transcricptase kinetics were performed in the absence or together with IPK1 and nevirapine (0.2 and 1 µM).
(TIF)
