# Supplementary figures and images for: Identification of a Novel Sulfonamide Non-Nucleoside Reverse Transcriptase Inhibitor by a Phenotypic HIV-1 Full Replication Assay
Source: PLoS One. 2013 Jul 18;8(7):e68767. doi: 10.1371/journal.pone.0068767 (PMC3715523; doi:10.1371/journal.pone.0068767)

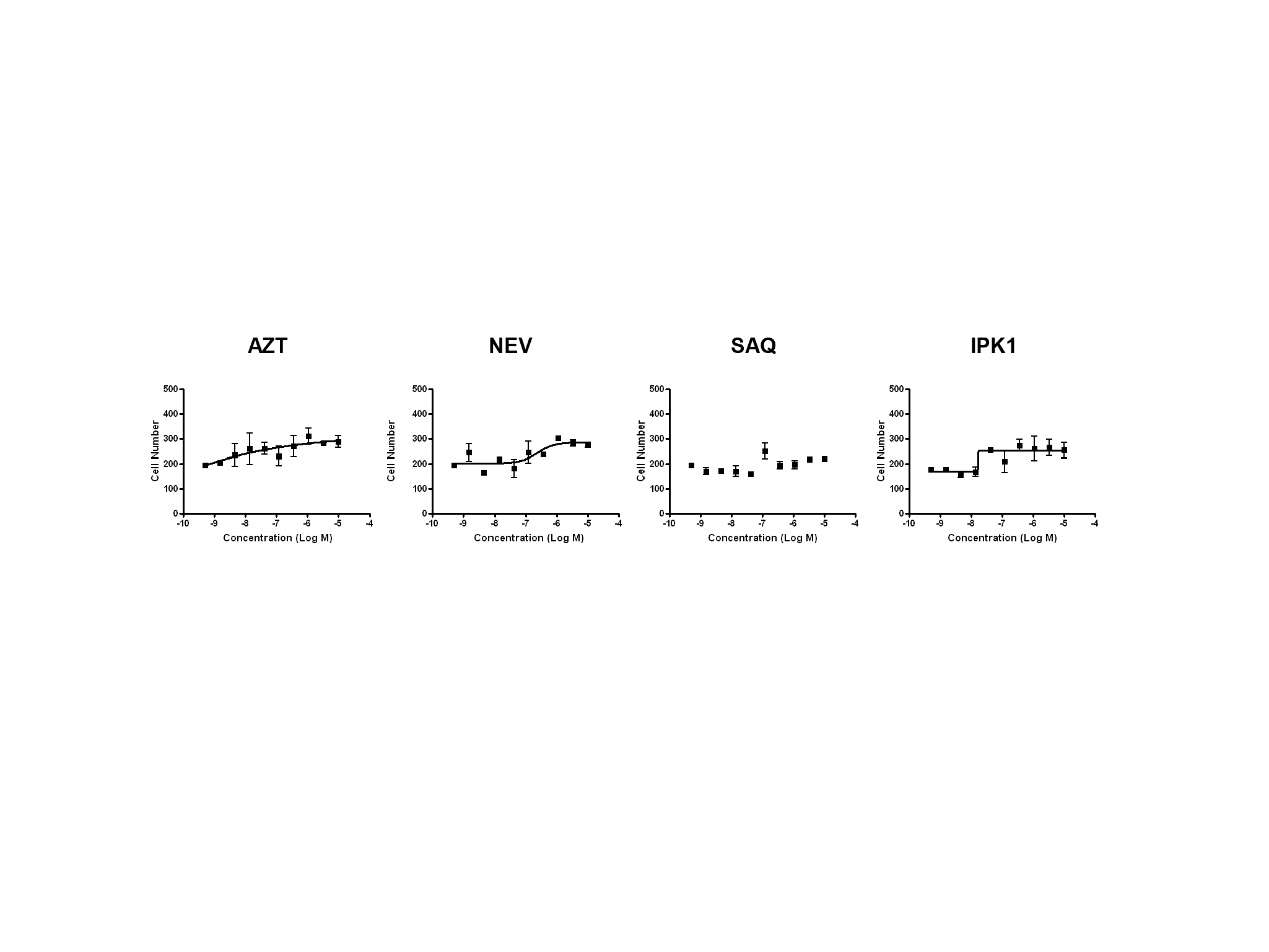

Supplement: Figure S1 — Determination of cellular toxicity by nucleus counting. Cells were incubated with several concentrations of IPK1, AZT, nevirapine, and saquinavir compounds. The nuclei of cells were stained by Syto60, and images were analyzed by Accapella Software. (TIF) [file pone.0068767.s001.tif]

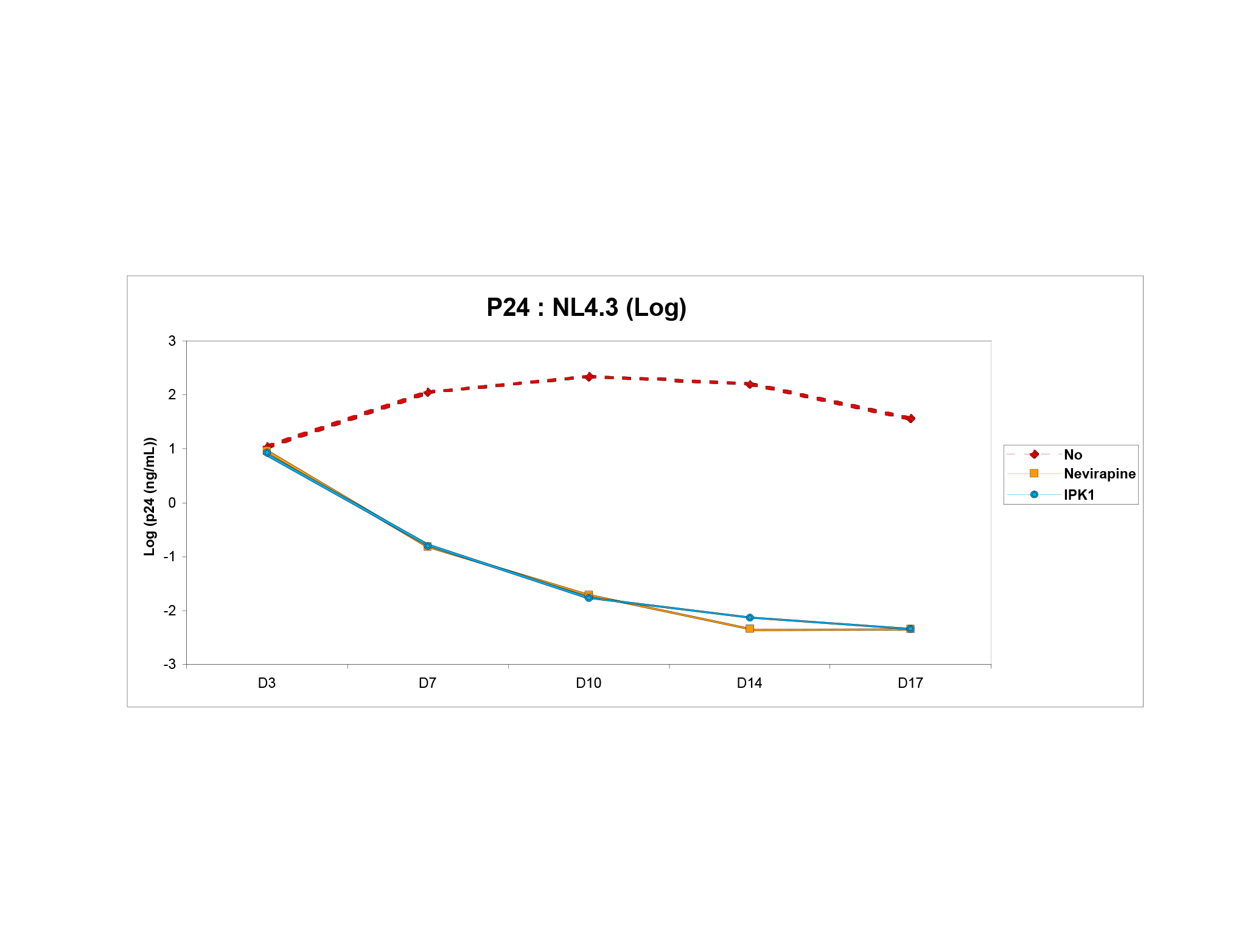

Supplement: Figure S2 — In vitro inhibition of viral replication. Antiviral IPK1 and nevirapine activity was analyzed using a p24 ELISA assay by measurement at day 3, 7, 10, 14 and 17 post-infection. (TIF) [file pone.0068767.s002.tif]

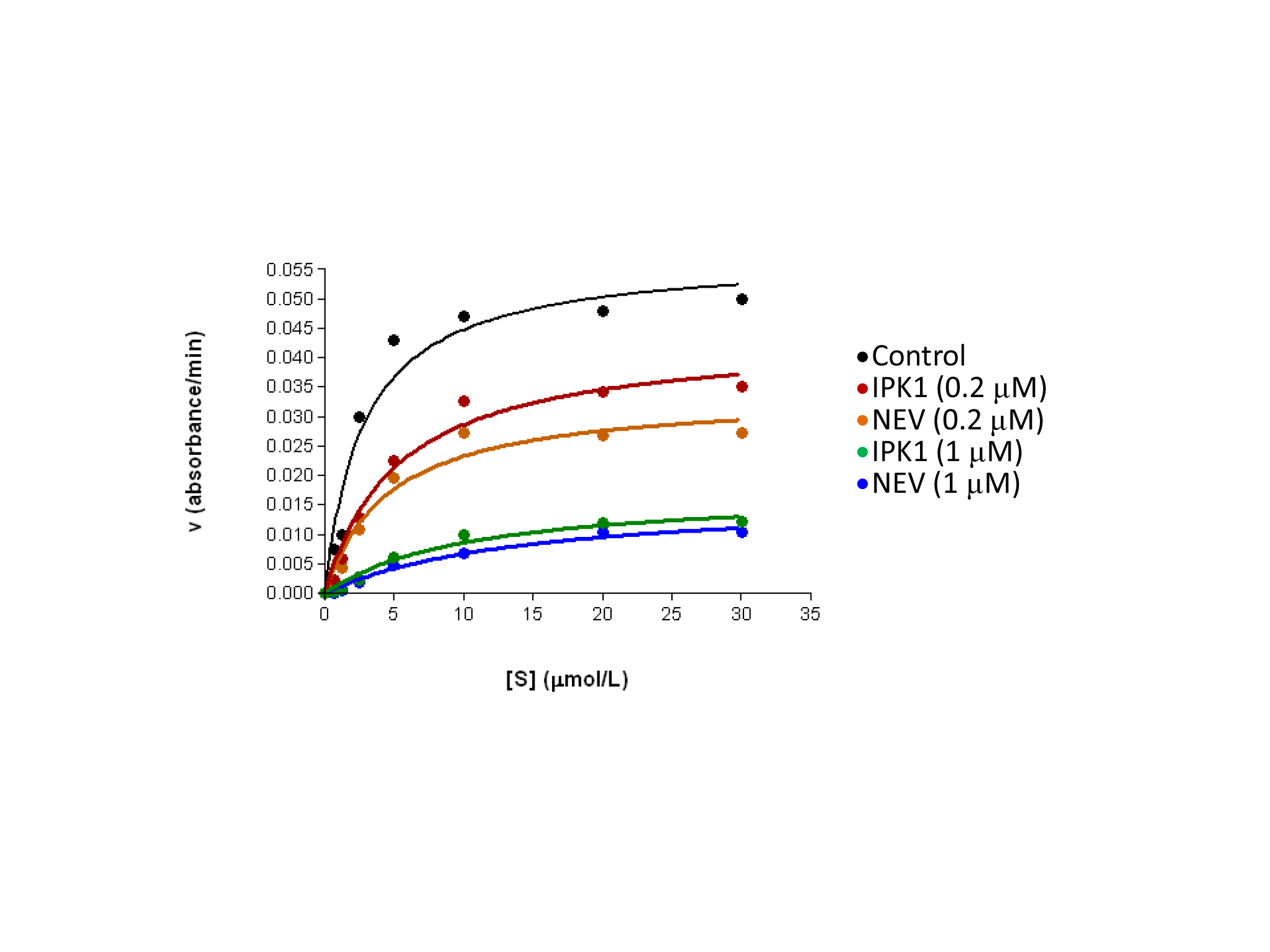

Supplement: Figure S3 — Kinetics of reverse transcriptase activity. Reverse transcricptase kinetics were performed in the absence or together with IPK1 and nevirapine (0.2 and 1 µM). (TIF) [file pone.0068767.s003.tif]
